# Gaps and Future Challenges of Italian Apps for Pregnancy and Postnatal Care: Systematic Search on App Stores

**DOI:** 10.2196/29151

**Published:** 2021-08-10

**Authors:** Laura Brunelli, Chiara De Vita, Fabrizio Cenedese, Michela Cinello, Marta Paris, Francesca Samogizio, Anja Starec, Michele Bava, Margherita Dal Cin, Sara Zanchiello, Tamara Stampalija

**Affiliations:** 1 Department of Medical, Surgical and Health Sciences University of Trieste Trieste Italy; 2 Quality and Accreditation Unit Friuli Centrale Healthcare University Trust Udine Italy; 3 Innovation and Complex Systems Department Area Science Park Trieste Italy; 4 Clinical Engineering, ICT and Procurement Institute for Maternal and Child Health, IRCCS Burlo Garofolo Trieste Italy; 5 Clinical Management Staff Institute for Maternal and Child Health, IRCCS Burlo Garofolo Trieste Italy; 6 Unit of Fetal Medicine and Prenatal Diagnosis Institute for Maternal and Child Health, IRCCS Burlo Garofolo Trieste Italy

**Keywords:** pregnancy, postnatal care, app, mHealth, mobile health, newborn

## Abstract

**Background:**

Despite the availability of thousands of health apps worldwide, when considering those addressing children’s first 1000 days of life, most apps fail to consider the continuity between the prenatal and postnatal stages, and their joint impact on maternal and child health. The reliability, quality, and effectiveness of these apps are largely unknown, and the provided content seems questionable in terms of completeness, updating, and trustworthiness.

**Objective:**

This study evaluates available Italian pregnancy and postnatal care apps to highlight the main gaps to be overcome and the resulting future challenges to be met in this mobile health–related field.

**Methods:**

A systematic search was conducted on the Apple App Store and Google Play Store, and basic information was collected for all identified apps. After deduplication and further selection based on the exclusion criteria, an in-depth analysis of each app was performed by two researchers independently. A 71-item six-domain questionnaire about the desirable features of apps was used to assess information, functionalities, and technical features, while the Mobile Application Rating Scale (MARS) was employed for app quality evaluation.

**Results:**

From an initial sample of 684 apps, 22 were deeply analyzed. Most apps did not fulfill the expectations, as just one achieved 50% of all desirable aspects. Postnatal care and counselling for both the mother and child was the least accomplished domain. Moreover, the quality of app information was generally rated more negatively than the quality of their functionality and esthetic features. The lacking aspects were information about methods for postpartum family planning and birth spacing (1/22, 5%) and immunization (2/22, 9%).

**Conclusions:**

The identified gaps could serve as a basis for designing and implementing increasingly high-quality, targeted, and effective apps for pregnancy and postnatal health care, which provide comprehensive, reliable, and evidence-based information, as well as appropriate esthetic and functional characteristics, with relevant implications in terms of maternal and newborn health prevention and promotion.

## Introduction

Recent development in information and communication technologies has enabled the disruptive expansion of electronic health (eHealth) and mobile health (mHealth). These developments, along with the introduction in clinical practice of technological innovations, such as telemedicine, telemonitoring, and remote screening, are considered essential elements of “game-changing innovations” in the next 25 years [1].

In fact, the widespread distribution of networked devices, which are estimated to reach 29.3 billion in 2023 [2], offers a promising but challenging opportunity of mHealth use for health information seeking, with an important role in health behavior formation [3-5]. In 2017, more than 325,000 health apps were available worldwide [6], and among them, to the best of our knowledge, there were more apps available to support pregnancy than for any other medical domain [7]. These mobile technologies in support of pregnancy have also increased the possibility for both parents and parents-to-be to self-manage health issues, as shown by a recent study conducted in 2019 in Switzerland reporting that 91% of parents declared using digital media for seeking information about their child’s health and development [8]. Moreover, a recent meta-analysis showed that social media and mHealth have the potential to be effective in promoting maternal physical health (eg, weight management), mental health, and knowledge about pregnancy [3]. However, when considering apps addressing children’s first 1000 days of life, from conception through age 24 months, many of them just focus on the prenatal or postnatal stage [9], failing to consider the continuity between the two phases and their joint impact on maternal and child health.

Furthermore, the reliability, quality, and effectiveness of current available pregnancy and postnatal care apps are unclear, and this could become an obstacle to health promotion, since during pregnancy women are more sensitive to external influences, and misleading information on health care and lifestyle could lead to unnecessary worrying or stress during pregnancy [10].

Considering the large variability among available apps in terms of property, responsibility of information accuracy, level of trustworthiness, and updating of content [11], as well as the lack of a certification system or their classification as a medical device [11,12], the limited evidence of their effectiveness is not a surprise [10]. In addition to this, standard app development and evaluation do not take into account the health literacy level of the target population [12,13], leaving to users the choice of mHealth to use, which therefore may be driven by popularity or esthetic, functional, and engagement aspects. In this regard, the literature suggests that many users do not critically assess the validity of the content provided by apps or consider issues concerning the privacy and security of their personal information and data [14].

Investigating previous research related to the impact of mHealth on maternal and child health care during pregnancy and in the postnatal period, the following two points have come to the forefront: (1) the consideration of children’s first 1000 days of life as a crucial developmental window for the children, where it is essential for mothers to receive accurate health information [3] and (2) the critical influence of the health information source on maternal well-being, lifestyle, and decision-making about pregnancy and child health during children’s first 1000 days of life [15]. Given these aspects and considering the striking gaps in the current literature, it is an ideal time to conduct an in-depth analysis on pregnancy and postnatal care apps currently in use.

The aim of this review was therefore to critically evaluate available Italian pregnancy and postnatal care apps and to highlight the main shortcomings and resulting future challenges about the apps for the health care of mothers and children in the first 1000 days of life.

## Methods

### Research on Italian Pregnancy and Postnatal Care Apps

A systematic search was conducted by six independent researchers from Trieste University, IRCCS (Istituto di Ricovero e Cura a Carattere Scientifico) Burlo Garofolo, and Area Science Park for the selection, data extraction, and functional evaluation of the available pregnancy and postnatal care apps on the Apple App Store and Google Play Store. The whole research team included public health specialists, psychologists, sociologists, clinical engineers, and a specialist in ergonomics (all under 45 years of age). The search was conducted for both the Apple App Store and Google Play Store using as keywords the Italian words for *pregnant*, *mother*, *9/nine months*, *birth*, *newborn*, *baby*, *obstetrics*, *pregnancy*, *new baby*, *my baby*, and *child* (the last four keywords were used with their English translation as well). The exclusion criteria for the research were as follows: gaming, photo and video, shopping, and commercial apps; calculator apps; apps specifically developed for health care professionals; fertility or menstrual tracking apps; and apps whose names were not available with Latin alphabets. A basic information set was collected for all resulting apps, including app name, operating system (iOS/Android), free/upon payment availability, availability of further contents upon payment, number of languages, availability of an Italian version, store category, dimension (MB), age restriction, number of downloads, position in download rankings, user rating, first release date, latest update, assistance responsibility, copyright, privacy policy, advertisement, and eventual presence of a medical device European mark (CE). The search was independently conducted between June 15 and July 3, 2020, by six researchers (three working on iOS and three on Android); any inconsistent information collected by the researchers was discussed until consensus was reached. Next, results were merged, and app deduplication was performed.

### In-Depth Analysis of Italian Pregnancy and Postnatal Care Apps

Further selection was made among identified apps according to additional exclusion criteria referring to apps not available in the Italian language and in both the Apple App Store and Google Play Store. The apps resulting from this selection were downloaded and installed on both iOS and Android devices for in-depth analysis. The six independent researchers were attributed the same number of apps each. For each app, two independent researchers registered and logged in to evaluate all app contents and functionalities, thus ensuring that each app would be evaluated on both the Apple App Store and Google Play Store. When relevant, a simulation of required input data, such as expected birth date and the starting date of the last menstrual period, was set to fully evaluate app potential. Pursuing this last aim, two evaluators simulated being in the first trimester of pregnancy, another two in the second trimester, and the last two in the third trimester to cover the entire pregnancy period. All investigators were able to consider and evaluate contents related to the postpartum period.

The analysis of the information, functionalities, and technical features of the apps was performed between September 1, 2020, and November 16, 2020, according to a 71-item assessment questionnaire investigating the desirable criteria of apps, developed based on the scientific literature [16], and multiprofessional discussion and agreement (Table 1).

As shown in Table 1, data items referred to six domains corresponding to pregnancy care and counselling, postnatal care and counselling for both the mother and child, reminders and push notifications, notes and records, social support, and app technical features. Each app was evaluated separately for all 71 questions. The answers were attributed as follows: yes (y) if the app provided the information/functionality/feature specified in the question; no (n) if the app did not provide the information/functionality/feature specified in the question; inconsistent information (i) in the case of inconsistent information derived from the operating system (iOS/Android) for the two researchers; and partially (p) if the app only partly provided the information/functionality/feature specified in the question. The Mobile Application Rating Scale (MARS) was used for app quality evaluation regarding four dimensions of objective app quality, including engagement, functionality, esthetics, and information quality. The subjective quality subscale and perceived impact section of the MARS were not included in the evaluation due to possible interresearched biases, as already reported in previous studies [17,18]. All MARS items were rated on a 5-point scale from 1 (inadequate) to 5 (excellent) and required the researcher to circle the number that most accurately represented the quality of the app component under evaluation. The means of the MARS scores judged by the six researchers were calculated for each app. Data were then merged and analyzed using Microsoft Excel for Office 365 ProPlus (Microsoft Corp). Fulfillment of the desirable criteria by each app was expressed as number and percentage on the specific domain and on the total of 71 questions. Responses to the questions by the group of apps was reported as number and percentage with descriptive statistics.

**Table 1:** Assessment questionnaire.

Domain	Questions
Pregnancy care and counselling	Does the app provide information about pregnancy? Does the app provide information about the woman’s rights during pregnancy (eg, at work, at school, and economical support)? Are prenatal risks and life-threatening conditions identified in the app? Does the app inform about maternal physiological and metabolic changes occurring during pregnancy? Does the app inform about the immunizations that the mother needs to receive? Does the app provide information about maternal or child services accessibility and contacts? Does the app provide information about available prenatal diagnostic tests? Does the app include physical exercises and workouts for women during pregnancy? Does the app provide pregnancy nutritional counselling for mothers? Does the app include personal hygiene practices for women during pregnancy? Does the app provide information about delivery? Does the app provide information about predelivery courses? Does the app provide month/trimester-related tips for pregnant women? Does the app provide a list of essentials for the hospital luggage? Does the app provide a list of free-of-charge and upon payment examinations during pregnancy?
Postnatal care and counselling for both mother and child	Does the app provide a list of essentials for the first welcome of the mother and baby at home? Does the app inform about maternal physiological and metabolic changes occurring during the postpartum period? Does the app include information about manifest neonatal complications and warning signs? Does the app offer information about postpartum mental disorders, such as postpartum depression and baby blues (eg, symptoms and coping strategies)? Does the app inform about the immunizations that mothers or newborns need during the first 1000 days? Does the app provide tips for the postpartum recovery process? Does the app provide practical tips on how to take care of the newborn (eg, hygiene, diapers’ changing, and burping)? Does the app provide postnatal nutritional counselling for mothers? Does the app provide a breastfeeding guide and support? Does the app report personal hygiene practices in the postnatal period? Does the app encompass methods for postpartum family planning and birth spacing?
Reminders and push notifications	Does the app allow to set reminders for medical appointments (eg, prenatal and postnatal check-ups, pediatric visits, and immunizations)? Does the app include push notification reminders for scheduled medications/immunizations? Does the app include push notification reminders when the pregnancy month/trimester begins? Does the app allow to schedule reminders for routine activities (eg, drinking, diapering, feeding, pumping, and sleeping)? Does the app allow users to change reminders and notifications settings?
Notes and records	Does the app require specifying the latest period date or the expected delivery date? Does the app allow the user to modify the expected delivery date following medical re-evaluation? Does the app allow to record physiological values of the mother (eg, pressure, temperature, and mood)? Does the app allow to record contractions or kicks? Does the app allow to record routine activities of the mother or the newborn (eg, drinking, steps, diapers changes, bottle feeding, and sleeping patterns/times)? Does the app allow to take note of the medical care the mother or the newborn has received (eg, medications and vaccination shots)? Does the app allow to track the newborn’s developmental milestones? Does the app record anthropometric measurements of the fetus (eg, height, weight, and head circumference)? Does the app record anthropometric measurements of the newborn (eg, height, weight, and head circumference)? Does the app record measurements of the mother’s weight at baseline and during pregnancy? Does the app record measurements of the mother’s weight in the postnatal period? Does the app allow the mother to create a sleep diary for herself? Does the app allow the mother to create a sleep diary for the newborn?
Social support	Is the app integrated with social networks (eg, Facebook and Twitter)? Is there a FAQ (frequently asked questions) page in the app? Does the app provide users with social mechanisms to interact with each other and share experiences (eg, community, forum, and chat)? Does the app provide users with social mechanisms to interact with health care staff (eg, community, forum, and chat)?
App technical features	Does the app ask users for authentication? Does the app present a privacy policy? If present, is that privacy policy properly written in Italian? Are all app contents freely available to the users (without any payment)? Are there specific inclusion criteria for full app usage (eg, national health service card, place of living, and certification by a health professional)? Does the app require to “sign” an informed consent for app usage? Does the app provide references about the provided contents? Does the app include a glossary of the most used medical terms? Does the app identify the scientific responsibility of the provided contents? Is there a possibility to back-up/restore data within the app? Is there a possibility to download data collected through the app? Does the app have multilanguage support? Does the app geolocalize the user to provide more detailed information? Does the app allow users to book visits, vaccinations, and checkups? Does the app allow users to update their account preferences? Does the app use a tone that is simple, informal, and friendly? Does the app adapt to screen orientation (both portrait and landscape)? Does the app learn user’s preferences over time? Does the app implement intuitive and predictable navigation patterns? Are the app contents validated by an institutional source (local, regional, or national)? Is the app a certified medical device according to Italian law? Does the app provide contents through different ways (eg, text, video, and audio)? Does the app ask about user satisfaction?

## Results

**Figure 1:**
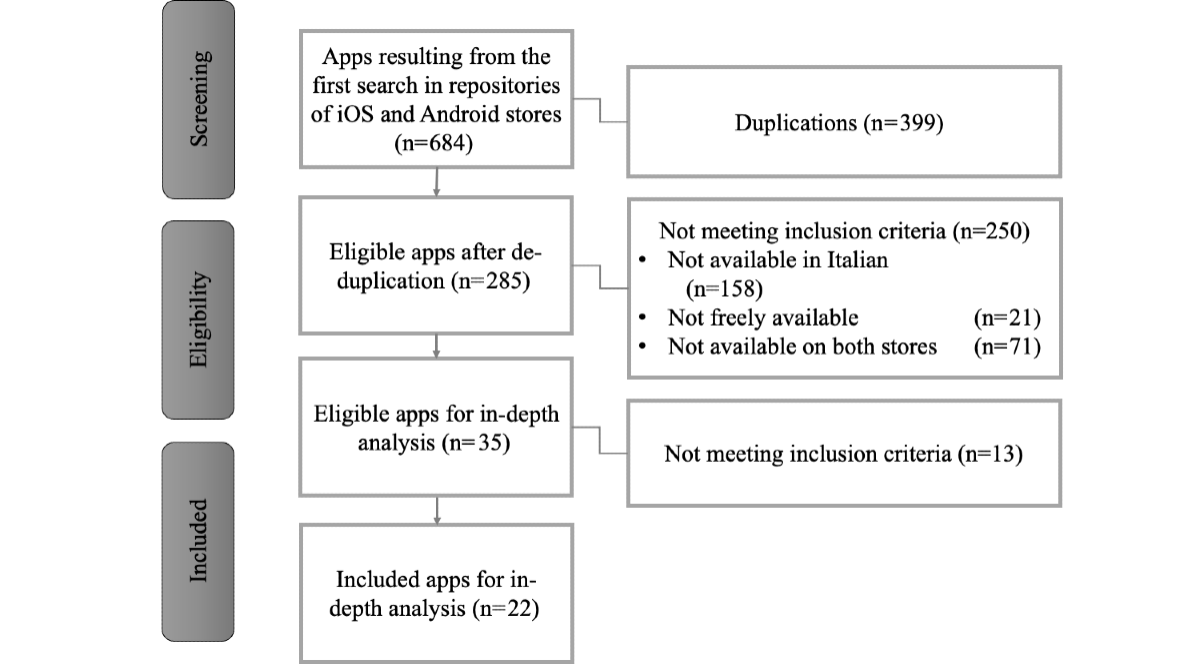
Results of the selection process of apps.

In total, 684 apps were initially identified; after deduplication, the number reduced to 285. Based on the exclusion criteria, 35 apps were downloaded and installed for in-depth analysis. The flow diagram illustrating the results of the selection process of apps is reported in Figure 1. Following the download of the apps and their further analysis, researchers excluded 13 additional apps as they met at least one of the exclusion criteria or had limitations in content or access that prevented their evaluation. In more detail, one app was not freely available, one was a photo and video app, two were only calculators, one was specifically developed for health professionals, two were menstrual tracking apps (one of which was the only app marked as a medical device among the 35 downloaded and installed apps), two were commercial apps, two contained only physical exercise suggestions, one contained information accessible through a premium account, and one did not work. At the end of the eligibility selection procedure, 22 Italian pregnancy and postnatal care apps underwent in-depth analysis.

There were 180 apps available in the Apple App Store and 148 available in the Google Play Store. Most of these apps (148/180, 82.2% and 146/148, 98.6% for iOS and Android, respectively) were freely available, but many offered extra content upon payment (95/180, 52.8% for iOS and 50/148, 33.8% for Android). Around half of them were available in Italian in both stores, and the majority presented any type of privacy policy. The distributions of the declared presence of advertisements within the app and some age usage restriction were quite different between the two stores. There were significant differences in dimensions among the two as well (Table 2). The mean user rating was well above the average value of 2.5 out of 5 in both stores, being greater in the Google Play Store. Finally, only one app was characterized by the presence of a medical device European mark (CE). The evaluated characteristics are reported in Table 2.

**Table 2:** Characteristics of the initial 285 apps for pregnancy and postnatal care identified after deduplication.

App characteristics	Apple App Store (n=180), n (%) or value	Google Play Store (n=148), n (%) or value
Freely available (yes)	148 (82.2%)	146 (98.6%)
Further content upon payment (yes)	95 (52.8%)	50 (33.8%)
Available in Italian (yes)	103 (57.2%)	62 (41.9%)
Privacy policy (yes)	141 (78.3%)	122 (82.4%)
Advertisement (yes)	10 (5.5%)	89 (60.1%)
Mean dimension (MB)	52.3	20.5
Some age restriction (yes)	106 (58.9%)	11 (7.4%)
Mean user rating (stars/5)	3.85	4.22
Medical device European mark (yes)	1	1

In Multimedia Appendix 1, data of in-depth analysis for the 22 evaluated apps are reported. Most apps did not fulfill the expectations, as just one met 50% of all desirable aspects while four met at least 40% of the expected requirements.

As reported in Table 3, the most accomplished domains were pregnancy care and counselling (124/330, 37.6%) and app technical features (160/506, 31.6%), followed by notes and records (77/286, 26.9%), social support (20/88, 22.7%), and postnatal care and counselling for both mother and child (54/242, 22.3%). The least accomplished domain was reminders and push notifications (24/110, 21.8%).

For pregnancy care and counselling, in addition to the general information about pregnancy provided in 14 (64%) cases, other information most frequently given included nutritional counselling for mothers (11/22, 50%), month/trimester-related tips for pregnant women (10/22, 46%), and information about delivery (10/22, 46%). Considering app technical features, a simple, informal, and friendly tone (22/22, 100%), and an intuitive and predictable navigation pattern (19/22, 86%) were the most frequently included features along with the presence of a privacy policy (17/22, 77%). Relating to the various domains, other aspects characterized at least half of the apps, including the presence of a properly written Italian privacy policy (13/22, 59%), as well as a frequently asked questions (FAQ) page (12/22, 55%), the requirement of the latest period date or the expected delivery date (12/22, 55%) and possibility of its eventual modification following medical re-evaluation (12/22, 55%), and the requirement of authentication for app usage (11/22, 50%).

**Table 3:** Level of accomplishment for each domain.

Domains	Yes^a^, n (%)	No^b^, n (%)	Inconsistent information^c^, n (%)	Partially^d^, n (%)
Pregnancy care and counselling (n=330)	124 (37.6%)	188 (57.0%)	3 (0.9%)	15 (4.5%)
Postnatal care and counselling for both mother and child (n=242)	54 (22.3%)	173 (71.5%)	3 (1.2%)	12 (5.0%)
Reminders and push notifications (n=110)	24 (21.8%)	83 (75.5%)	2 (1.8%)	1 (0.9%)
Notes and records (n=286)	77 (26.9%)	200 (69.9%)	0 (0.0%)	9 (3.1%)
Social support (n=88)	20 (22.7%)	67 (76.1%)	0 (0.0%)	1 (1.1%)
App technical features (n=506)^e^	160 (31.6%)	329 (65.0%)	14 (2.8%)	1 (0.2%)

^a^If the app provided the information/functionality/feature.

^b^If the app did not provide the information/functionality/feature.

^c^In the case of inconsistent information derived from the operating system (iOS/Android) of the two researchers.

^d^If the app only partly provided the information/functionality/feature.

^e^Not applicable for two apps.

Aspects that were particularly lacking included information about methods for postpartum family planning and birth spacing (1/22, 5%), immunizations that mothers or newborns need to be administered (2/22, 9%), and postnatal personal hygiene practices for the mother (2/22, 9%). Notably, information about free-of-charge and upon payment examinations during pregnancy was present in only 14% (3/22) of apps, but partial information was given by 32% (7/22) of apps.

Few apps have push notification reminders for scheduled medications/immunizations (3/22, 14%) or when the pregnancy month/trimester begins (3/22, 14%), and a minority of them allow mothers to schedule reminders for routine activities (2/22, 9%) or to take notes of medical care received by both the mother and newborn (2/22, 9%), as well as to monitor the mother’s sleep (1/22, 5%). Furthermore, a limited number of apps allow users to record physiological values of the mother (3/22, 14%), routine activities of both the mother and newborn (3/22, 14%), or the newborn’s developmental milestones (3/22, 14%). Almost all apps do not provide users with social mechanisms to interact and share experiences with each other (21/22, 96%) or with the health care staff (21/22, 96%). Regarding app technical features, only in a few cases, data collected through the app can be downloaded (3/22, 14%), and a minority of apps have multilanguage support (3/22, 14%) or geolocalize the user to provide more detailed information (3/22, 14%). Just one app requires the habilitation of the pregnant women by the regional health care system as a specific inclusion criterion (1/22, 5%). Only two apps learn user preferences over time (2/22, 9%), but none of them directly allows the mother to book visits, vaccinations, or checkups. Moreover, no app is marked as a medical device according to the medical device directive 93/42/EEC, and subsequent modifications and integrations.

The MARS scores for global app quality ranged from a minimum score of 1.9 to a maximum of 4.5, with most apps (16/22, 73%) achieving a score greater than 3. The engagement score ranged from 1.6 to 4.1 (median 3.0), functionality score ranged from 2.5 to 4.9 (median 4.0), esthetics score ranged from 1.8 to 4.3 (median 3.7), and information score ranged from 1.7 to 4.8 (median 3.5). The mean MARS scores for all 22 analyzed apps are reported in Multimedia Appendix 2.

## Discussion

### Principal Findings

This systematic search aimed to evaluate the available Italian pregnancy and postnatal care apps, providing an overview of their main characteristics with specific focuses on shortcomings and gaps to be filled with future developments in the eHealth-related field. We believe that such an assessment is indeed crucial for the implementation of increasingly effective apps for pregnancy and postnatal health care. Overall, the presence of a single app containing more than half of the investigated desirable aspects suggests considerable room for improvement in developing apps targeted at the needs of pregnant women and new mothers.

Since during pregnancy women are more sensitive to misleading information [10] and considering the potential of mHealth to be effective in promoting maternal health and knowledge about pregnancy and child health and development [3,8], the importance of developing apps able to provide the most complete, truthful, and reliable information on pregnancy and the postnatal period is unquestionable. In this regard, less than a third of the analyzed apps provided references about included contents or identified their scientific responsibility. Furthermore, the quality of information included in the examined apps was rated more negatively than the quality of their functionality and esthetic features. This aspect can prove to be a double-edged sword, since, as suggested in the literature, the choice of mHealth to be used may be driven by popularity, esthetic features, and functional features rather than the validity, veracity, and reliability of the provided content [14]. Similarly, the use of a simple, informal, and friendly tone, as well as the presence of intuitive and predictable navigation patterns, which are the most frequently included features in the analyzed apps, may pose a threat. While these aspects make an app intuitive, user friendly, and pleasant to use, they may also attract attention and prompt mothers and mothers-to-be to use the app even in the absence of reliable health-related information. In this regard, the tendency to be driven by esthetic or engagement aspects could be particularly detrimental to mothers with low health literacy who lack critical capacity and awareness of the information they receive [12,13]. The development of apps that are both esthetically pleasing and sources of accurate, valid, and comprehensive information, providing indications about the reliability of content, is therefore highly desirable [19].

Moreover, the fact that no app is marked as a medical device denotes a serious shortcoming, which needs to be addressed through the development of apps that are not only information or entertainment tools, but also the subject of a specific regulatory regime, the new medical device regulation. The latter underlines the importance of medical device apps in terms of their predictive utilization for mothers and children, and the control or support of conception.

Regarding provided content, postnatal care and counselling for both the mother and child represented the thematic domain least covered by the analyzed apps, pointing out that most of the available apps focus on pregnancy and neglect the postpartum period. It would therefore be advisable for an app to provide more balanced information on the stages of the mother’s life and newborn infant’s life, thus paying attention to both prenatal and postnatal care. This point is in line with the literature suggesting that most of the currently available apps just focus on the prenatal or postnatal stage [9], neglecting the continuity between the two phases and their joint impact on the health of both the mother and child.

Surprisingly, information about free-of-charge and upon payment clinical examinations during pregnancy and about immunizations that mothers and newborns need to receive is absent in most apps. This highlights a gap that should be filled through new mobile apps that encourage the promotion of disease prevention for mothers, newborns, and infants and facilitate early intervention in case of problems or complications during pregnancy. An app that provides correct and up-to-date medical information related to preventive or diagnostic medical practices, such as immunizations and examinations, can be a tool to support the health care system by guiding women to the most appropriate screening and therapeutic paths.

Moreover, most of the evaluated apps do not seem to encourage interaction, communication, and confrontation of pregnant women either with other women in the same condition or with health professionals, not taking into consideration the dimension of sociability and the importance of support from both peers and competent professionals. This deficiency, especially in certain periods (eg, during the COVID-19 pandemic), could be burdensome for the woman, potentially generating a sense of loneliness and disorientation.

Very few apps include push notification reminders for scheduled medical appointments, and a minority of apps allow mothers to schedule reminders for routine activities. It follows that the examined apps are not particularly interactive, giving mothers and mothers-to-be few opportunities to rely on them as reference and guidance tools throughout the day or during the pregnancy and postnatal periods. Furthermore, no app directly allows the mother to book visits, vaccinations, or checkups, highlighting the absence of a direct link between the app and reservation systems, and therefore, a lack of integration of this tool within the wider public national or regional health care system. This major gap could be filled by the development of more interconnected and institutional apps that could streamline booking procedures by reducing the number of phone calls or other request modes from patients and the staff involved in managing them.

Furthermore, only two apps could learn user preferences over time, denoting a poor ability of the analyzed apps to adapt to the type of user. Greater “intelligence” of the tool and the consequent possibility of greater customization and profiling of the app system would be desirable, thus resulting in a more tailored option for each woman.

Longitudinal studies, focused on the use of apps by mothers and mothers-to-be over time, could be very useful to highlight which technical features, functionalities, and contents of apps (and to what extent) may be most effective in promoting pregnancy and postnatal health care. The results of such longitudinal research may provide evidence-based guidelines based on which increasingly effective apps could be developed and implemented, thus becoming effective tools for improving health.

### Limitations

This study has some limitations, which include only analysis of apps available in Italian and available on both the Apple App Store and Google Play Store without charge. Even if this prevented the researchers from including all apps, this choice was driven by the need to analyze apps with a potentially large user base aiming at equity and equality, and the need for researchers to fully understand the content proposed by the apps. Second, we did not directly involve pregnant women or mothers during the postnatal period as evaluators. Third, even if developed following previous research available in the scientific literature, the definitions of the 71 items for the app evaluation were in part the result of original work of multiprofessional comparison and agreement based on the experience and expertise of the research team. Finally, considering the nature of the dimensions investigated by the MARS, subjectivity in awarding its scores cannot be ruled out. Nevertheless, our research team included members under 45 years of age (mostly women), and some of the women had been pregnant; therefore, we can assume that no biases were there for the assessment procedure. Moreover, in the case of one app, we were prevented from accessing full contents and features, so our evaluation could present some undesired bias in this specific case.

### Conclusion

People are increasingly using the internet and specific apps to find health information thanks to the ease of access and the speed of communication that digital solutions offer. While this creates a wide range of advantages and new opportunities, it can pose a threat to public ​​health in terms of prevention and promotion, with exposure to risks, such as lack of content control and misinformation. Within this framework, this study aimed to highlight the main shortcomings in available Italian pregnancy and postnatal care apps to serve as a basis for designing and implementing increasingly high-quality apps that provide comprehensive, reliable, and evidence-based information, and have appropriate esthetic and functional characteristics. Further developments are desirable in the eHealth-related field, with a view to encourage increasingly conscious and effective use of apps by mothers and mothers-to-be for pregnancy and postnatal care.

## Appendix

Multimedia Appendix 1 Total number and percentage of accomplishments for each questionnaire item and globally for each of the 22 apps.

Multimedia Appendix 2 Mobile Application Rating Scale scores for the 22 evaluated apps.

## Abbreviations

MARS: Mobile Application Rating Scale
