# Supplementary material for: Gaps and Future Challenges of Italian Apps for Pregnancy and Postnatal Care: Systematic Search on App Stores
Source: J Med Internet Res. 2021 Aug 10;23(8):e29151. doi: 10.2196/29151 (PMC8386367; doi:10.2196/29151)
Supplement: Multimedia Appendix 1 [file jmir_v23i8e29151_app1.docx]

**Multimedia Appendix 1.** Total number and percentage of accomplishments for each questionnaire item and globally for each of the 22 apps.

| Domain | Questions | Bebe+ | iMamma | La mia gravidanza (Aleksei Neiman) | Autosvezzamento ricette veloci | Gravidanza+ | Mamma 2.0 | Vera mamma | | Yoga in Gravidanza: la guida | | VITA: prodotti in gravidanza | Dalla nascita | Gravidanza Sprout | Mamma in salute | Calendario WomanLog Baby | | La mia gravidanza (Doctissimo) | Non Da Sola* (Emilia Romagna Region-specific) | iBimbo | SOS bimbi | happy mamma * (Toscana Region-specific) | mustela per me | seimammaeuganea | pregnancy | eMyBaby | Tot y  (n; %) | Tot n  (n; %) | Tot i  (n; %) | Tot p  (n; %) |  |
| --- | --- | --- | --- | --- | --- | --- | --- | --- | --- | --- | --- | --- | --- | --- | --- | --- | --- | --- | --- | --- | --- | --- | --- | --- | --- | --- | --- | --- | --- | --- | --- |
| Pregnancy care and counselling | 1 | n | y | n | n | y | n | y | | n | | y | y | y | y | n | | y | y | y | n | y | y | y | y | n | 14; 3.6 | 8; 36.4 | 0; 0.0 | 0; 0.0 |  |
|  | 2 | n | y | n | n | n | n | n | | n | | n | y | n | n | n | | y | y | n | n | y | y | y | n | n | 7; 31.8 | 15; 8.2 | 0; 0.0 | 0; 0.0 |  |
|  | 3 | n | n | n | n | y | n | n | | n | | y | n | y | y | n | | y | y | n | n | y | y | y | n | n | 9; 40.9 | 13; 9.1 | 0; 0.0 | 0; 0.0 |  |
|  | 4 | n | y | n | n | y | n | y | | n | | y | n | y | y | n | | y | y | n | n | n | y | n | n | n | 9; 40.9 | 13; 9.1 | 0; 0.0 | 0; 0.0 |  |
|  | 5 | n | p | p | n | n | n | n | | n | | n | n | p | y | n | | y | y | n | n | y | n | n | n | n | 4; 18.2 | 15; 8.2 | 0; 0.0 | 3; 13.6 |  |
|  | 6 | n | n | n | n | n | n | n | | n | | n | y | n | y | n | | n | y | n | p | y | n | y | n | n | 5; 22.7 | 16; 2.7 | 0; 0.0 | 1; 4.5 |  |
|  | 7 | n | y | n | n | y | n | y | | n | | n | y | y | y | n | | y | y | n | n | y | p | i | n | n | 9; 40.9 | 11; 0.0 | 1; 4.5 | 1; 4.5 |  |
|  | 8 | n | y | n | n | y | n | y | | y | | y | n | n | n | n | | y | n | n | n | y | y | n | n | n | 8; 36.4 | 14; 3.6 | 0; 0.0 | 0; 0.0 |  |
|  | 9 | n | y | n | n | y | n | y | | n | | y | y | y | y | n | | y | y | n | n | y | y | n | n | n | 11; 0.0 | 11; 0.0 | 0; 0.0 | 0; 0.0 |  |
|  | 10 | n | y | n | n | y | n | n | | n | | y | y | n | y | n | | y | n | n | n | n | y | y | n | n | 8; 36.4 | 14; 3.6 | 0; 0.0 | 0; 0.0 |  |
|  | 11 | n | y | n | n | y | n | n | | n | | y | y | y | y | n | | y | y | n | n | y | p | y | n | n | 10; 5.5 | 11; 5.0 | 0; 0.0 | 1; 4.5 |  |
|  | 12 | n | y | n | n | y | n | n | | n | | n | y | y | y | n | | n | y | n | n | y | i | y | n | n | 8; 36.4 | 13; 9.1 | 1; 4.5 | 0; 0.0 |  |
|  | 13 | n | y | y | n | y | n | y | | n | | y | n | y | y | n | | y | p | n | n | n | y | y | n | n | 10; 5.5 | 11; 0.0 | 0; 0.0 | 1; 4.5 |  |
|  | 14 | n | y | y | n | y | n | y | | n | | y | y | y | y | n | | y | n | n | n | n | p | n | n | n | 9; 40.9 | 12; 4.5 | 0; 0.0 | 1; 4.5 |  |
|  | 15 | n | y | p | n | p | n | p | | n | | n | p | p | y | n | | p | y | n | n | p | n | i | n | n | 3; 13.6 | 11; 0.0 | 1; 4.5 | 7; 31.8 |  |
| Postnatal care and counselling for both mother and child | 16 | n | n | y | n | n | n | y | | n | | y | n | y | n | n | | y | p | n | n | n | n | n | n | n | 5; 22.7 | 16; 2.7 | 0; 0.0 | 1; 4.5 |  |
|  | 17 | y | n | n | n | y | n | n | | n | | y | n | n | p | n | | y | p | n | n | y | n | y | n | n | 6; 27.3 | 14; 3.6 | 0; 0.0 | 2; 9.1 |  |
|  | 18 | n | n | n | n | n | n | n | | n | | y | n | n | y | n | | y | n | n | y | y | n | n | n | n | 5; 22.7 | 17; 7.3 | 0; 0.0 | 0; 0.0 |  |
|  | 19 | y | n | n | n | y | n | y | | n | | y | n | y | y | n | | y | y | n | n | y | n | i | n | n | 9; 40.9 | 12; 4.5 | 1; 4.5 | 0; 0.0 |  |
|  | 20 | p | n | n | n | n | n | p | | n | | n | n | n | y | n | | p | p | n | p | y | n | n | n | n | 2; 9.1 | 15; 8.2 | 0; 0.0 | 5; 22.7 |  |
|  | 21 | p | n | n | n | n | n | y | | n | | y | n | n | n | n | | y | n | n | n | n | n | y | n | n | 4; 18.2 | 17; 7.3 | 0; 0.0 | 1; 4.5 |  |
|  | 22 | y | n | n | n | y | n | y | | n | | y | n | n | y | y | | n | n | n | n | n | n | n | n | n | 6; 27.3 | 16; 2.7 | 0; 0.0 | 0; 0.0 |  |
|  | 23 | n | n | n | n | n | y | y | | n | | y | y | n | n | n | | y | n | n | n | n | n | n | n | n | 5; 22.7 | 17; 7.3 | 0; 0.0 | 0; 0.0 |  |
|  | 24 | y | n | n | n | y | n | n | | n | | y | y | y | y | n | | y | y | n | y | i | n | p | n | n | 9; 40.9 | 11; 0.0 | 1; 4.5 | 1; 4.5 |  |
|  | 25 | n | n | n | n | n | n | n | | n | | p | y | n | n | n | | y | n | n | p | n | n | n | n | n | 2; 9.1 | 18; 1.8 | 0; 0.0 | 2; 9.1 |  |
|  | 26 | n | n | n | n | n | n | n | | n | | y | n | n | n | n | | n | n | n | n | i | n | n | n | n | 1; 4.5 | 20; 0.9 | 1; 4.5 | 0; 0.0 |  |
| Reminders and push notifications | 27 | y | y | n | n | n | n | | n | | n | y | y | y | y | n | p | | n | n | n | n | y | y | n | y | 9; 40.9 | 12; 4.5 | 0; 0.0 | 1; 4.5 |  |
|  | 28 | n | y | n | n | n | n | n | | n | | n | n | n | n | n | | y | n | n | n | n | y | n | n | n | 3; 13.6 | 19: 6.4 | 0; 0.0 | 0; 0.0 |  |
|  | 29 | n | y | y | n | n | n | n | | n | | n | n | n | n | n | | n | n | n | n | n | y | n | n | n | 3; 13.6 | 19; 6.4 | 0; 0.0 | 0; 0.0 |  |
|  | 30 | n | n | n | n | n | n | n | | n | | n | n | i | y | n | | n | n | n | n | n | y | n | n | n | 2; 9.1 | 19; 6.4 | 1; 4.5 | 0; 0.0 |  |
|  | 31 | y | y | y | n | y | n | n | | n | | n | n | n | n | y | | n | n | n | n | i | y | n | y | n | 7; 31.8 | 14; 3.6 | 1; 4.5 | 0; 0.0 |  |
| Notes and records | 32 | n | y | y | n | y | n | y | | n | | n | n | y | y | n | | y | y | n | n | n | y | y | y | y | 12; 4.5 | 10; 5.5 | 0; 0.0 | 0; 0.0 |  |
|  | 33 | n | y | y | n | y | n | y | | n | | y | n | y | y | n | | y | n | y | n | n | y | y | y | n | 12; 4.5 | 10; 5.5 | 0; 0.0 | 0; 0.0 |  |
|  | 34 | n | y | n | n | n | n | n | | n | | n | n | n | p | n | | n | n | n | n | n | y | n | n | y | 3; 13.6 | 18; 1.8 | 0; 0.0 | 1; 4.5 |  |
|  | 35 | n | y | y | n | y | n | y | | n | | n | n | y | y | n | | y | n | n | n | n | n | n | y | y | 9; 40.9 | 13; 9.1 | 0; 0.0 | 0; 0.0 |  |
|  | 36 | p | p | n | n | n | n | n | | n | | p | n | n | p | y | | n | n | y | n | n | y | n | n | n | 3; 13.6 | 15; 8.2 | 0; 0.0 | 4; 18.2 |  |
|  | 37 | p | y | n | n | n | n | n | | n | | n | n | n | n | n | | n | n | n | n | n | y | n | n | n | 2; 9.1 | 19; 6.4 | 0; 0.0 | 1; 4.5 |  |
|  | 38 | y | n | n | n | n | n | n | | n | | n | n | n | n | y | | n | n | p | n | n | y | n | n | n | 3; 13.6 | 18; 1.8 | 0; 0.0 | 1; 4.5 |  |
|  | 39 | n | p | y | n | p | n | y | | n | | y | n | n | n | n | | n | n | n | n | n | y | n | y | y | 6; 27.3 | 14; 3.6 | 0; 0.0 | 2; 9.1 |  |
|  | 40 | y | n | n | n | n | n | n | | n | | n | n | n | n | y | | n | n | y | n | n | y | n | n | y | 5; 22.7 | 17; 7.3 | 0; 0.0 | 0; 0.0 |  |
|  | 41 | n | y | y | n | y | n | y | | n | | n | n | y | y | n | | y | n | n | n | n | y | n | y | y | 10; 5.5 | 12; 4.5 | 0; 0.0 | 0; 0.0 |  |
|  | 42 | y | y | y | n | y | n | y | | n | | n | n | y | n | n | | n | n | n | n | n | y | n | n | n | 7; 31.8 | 15; 8.2 | 0; 0.0 | 0; 0.0 |  |
|  | 43 | n | n | n | n | n | n | n | | n | | n | n | n | n | n | | n | n | n | n | n | y | n | n | n | 1; 4.5 | 21; 5.5 | 0; 0.0 | 0; 0.0 |  |
|  | 44 | y | n | n | n | n | n | n | | n | | n | n | n | n | y | | n | n | y | n | n | y | n | n | n | 4; 18.2 | 18; 1.8 | 0; 0.0 | 0; 0.0 |  |
| Social support | 45 | n | y | n | y | n | y | n | | n | | n | n | y | y | n | | n | y | n | n | n | n | n | n | n | 6; 27.3 | 16; 2.7 | 0; 0.0 | 0; 0.0 |  |
|  | 46 | y | y | n | n | y | n | y | | n | | n | p | n | y | n | | y | y | y | y | y | y | n | y | n | 12; 4.5 | 9; 40.9 | 0; 0.0 | 1: 4.5 |  |
|  | 47 | n | y | n | n | n | n | n | | n | | n | n | n | n | n | | n | n | n | n | n | n | n | n | n | 1; 4.5 | 21; 5.5 | 0; 0.0 | 0; 0.0 |  |
|  | 48 | n | n | n | n | n | n | n | | n | | n | n | n | n | n | | n | n | n | n | n | n | y | n | n | 1; 4.5 | 21; 5.5 | 0; 0.0 | 0; 0.0 |  |
| App technical features | 49 | y | y | n | n | y | y | y | | n | | y | n | n | n | y | | n | n | i | n | y | y | n | y | y | 11; 0.0 | 10; 5.5 | 1; 4.5 | 0; 0.0 |  |
|  | 50 | y | y | y | y | y | y | y | | y | | y | n | n | n | y | | y | y | y | n | y | y | y | n | y | 17; 7.3 | 5; 22.7 | 0; 0.0 | 0; 0.0 |  |
|  | 51 | y | y | y | y | y | n | y | | y | | n | n | n | n | n | | n | y | y | NA | y | y | y | NA | y | 13; 9.1 | 7; 31.8 | 0; 0.0 | 0; 0.0 |  |
|  | 52 | y | y | y | n | n | y | y | | n | | n | y | i | y | n | | y | y | i | y | y | y | y | n | y | 14; 3.6 | 6; 27.3 | 2; 9.1 | 0; 0.0 |  |
|  | 53 | n | n | n | n | n | n | n | | n | | n | n | n | n | n | | n | n | n | n | y | n | n | n | n | 1; 4.5 | 21; 5.5 | 0; 0.0 | 0; 0.0 |  |
|  | 54 | n | y | y | n | y | n | n | | n | | n | n | n | n | n | | y | y | n | n | i | n | n | n | y | 6; 27.3 | 15; 8.2 | 1; 4.5 | 0; 0.0 |  |
|  | 55 | n | n | n | n | n | n | n | | n | | n | y | n | y | n | | y | y | n | y | y | n | n | n | n | 6; 27.3 | 16; 2.7 | 0; 0.0 | 0; 0.0 |  |
|  | 56 | n | n | n | n | n | n | n | | n | | n | y | n | y | n | | y | n | n | p | y | n | n | n | n | 4; 18.2 | 17; 7.3 | 0; 0.0 | 1; 4.5 |  |
|  | 57 | n | n | n | n | n | n | n | | n | | n | n | n | y | n | | n | y | n | y | y | n | y | n | n | 5; 22.7 | 17; 7.3 | 0; 0.0 | 0; 0.0 |  |
|  | 58 | y | n | n | n | y | n | n | | n | | n | n | n | n | y | | n | n | i | n | n | n | n | y | y | 5; 22.7 | 16; 2.7 | 1; 4.5 | 0; 0.0 |  |
|  | 59 | y | n | n | n | y | n | n | | n | | n | n | n | n | y | | n | n | n | n | n | n | n | n | n | 3; 13.6 | 19; 6.4 | 0; 0.0 | 0; 0.0 |  |
|  | 60 | n | n | n | y | n | n | n | | n | | n | n | n | n | i | | n | i | n | n | n | n | n | y | y | 3; 13.6 | 17; 7.3 | 2; 9.1 | 0; 0.0 |  |
|  | 61 | n | y | n | n | n | n | n | | n | | n | n | n | n | n | | n | y | n | y | i | n | n | n | n | 3; 13.6 | 18; 1.8 | 1; 4.5 | 0; 0.0 |  |
|  | 62 | n | n | n | n | n | n | n | | n | | n | n | n | n | n | | n | n | n | n | n | n | n | n | n | 0; 0.0 | 22; 100 | 0; 0.0 | 0; 0.0 |  |
|  | 63 | y | y | y | n | y | n | n | | n | | y | n | n | n | y | | y | n | i | n | n | y | n | y | n | 9; 40.9 | 12; 4.5 | 1; 4.5 | 0; 0.0 |  |
|  | 64 | y | y | y | y | y | y | y | | y | | y | y | y | y | y | | y | y | y | y | y | y | y | y | y | 22; 100 | 0; 0.0 | 0; 0.0 | 0; 0.0 |  |
|  | 65 | n | n | n | i | n | y | n | | y | | n | n | i | i | y | | n | n | n | n | n | n | n | y | n | 4; 18.2 | 15; 8.2 | 3; 13.6 | 0; 0.0 |  |
|  | 66 | n | y | n | n | n | n | n | | n | | y | n | n | n | n | | n | n | n | n | n | n | n | n | n | 2; 9.1 | 20; 0.9 | 0; 0.0 | 0; 0.0 |  |
|  | 67 | y | y | y | y | y | y | y | | y | | n | y | y | y | n | | y | n | y | y | y | y | y | y | y | 19; 6.4 | 3; 13.6 | 0; 0.0 | 0; 0.0 |  |
|  | 68 | n | n | n | n | n | n | n | | n | | n | n | n | y | n | | n | y | n | y | y | n | y | n | n | 5; 22.7 | 17; 7.3 | 0; 0.0 | 0; 0.0 |  |
|  | 69 | n | n | n | n | n | n | n | | n | | n | n | n | n | n | | n | n | n | n | n | n | n | n | n | 0; 0.0 | 22; 100 | 0; 0.0 | 0; 0.0 |  |
|  | 70 | n | y | n | n | n | n | n | | y | | n | n | i | n | n | | n | y | n | y | n | n | n | n | n | 4; 18.2 | 17; 7.3 | 1; 4.5 | 0; 0.0 |  |
|  | 71 | y | n | i | n | y | n | n | | n | | n | n | y | n | y | | n | n | n | n | n | n | n | n | n | 4; 18.2 | 17; 7.3 | 1; 4.5 | 0; 0.0 |  |
| tot y (n;  %) | | 21;  29.6 | 37;  52.1 | 18;  25.4 | 6;  8.5 | 32;  45.1 | 8;  11.3 | 25;  35.2 | | 7;  9.9 | | 26;  36.6 | 18;  25.4 | 22;  31.0 | 33;  46.5 | 14;  19.7 | | 34;  47.9 | 26;  36.6 | 10;  14.1 | 11;  15.5 | 26;  36.6 | 33;  46.5 | 21;  29.6 | 15;  21.1 | 16;  22.5 |  | | | | |
| tot n (n;  %) | | 46;  64.8 | 31;  43.7 | 50;  70.4 | 64;  90.1 | 37;  52.1 | 63;  88.7 | 44;  62.0 | | 64;  90.1 | | 43;  60.6 | 51;  71.8 | 43;  60.6 | 34;  47.9 | 56;  78.9 | | 34;  47.9 | 40;  56.3 | 56;  78.9 | 55;  77.5 | 39;  54.9 | 34;  47.9 | 46;  64.8 | 55;  77.5 | 55;  77.5 |  |  |  |  |  |
| tot p (n;  %) | | 4; 5.6 | 3;  4.2 | 2;  2.8 | 0;  0.0 | 2;  2.8 | 0;  0.0 | 2;  2.8 | | 0;  0.0 | | 2;  2.8 | 2;  2.8 | 2;  2.8 | 3;  4.2 | 0;  0.0 | | 3;  4.2 | 4;  5.6 | 1;  1.4 | 4;  5.6 | 1;  1.4 | 3;  4.2 | 1;  1.4 | 0;  0.0 | 0;  0.0 |  |  |  |  |  |

*Note.* y=yes; n=no; p=partially; i=inconsistent information; NA=not applicable
