# Supplementary material for: Gaps and Future Challenges of Italian Apps for Pregnancy and Postnatal Care: Systematic Search on App Stores
Source: J Med Internet Res. 2021 Aug 10;23(8):e29151. doi: 10.2196/29151 (PMC8386367; doi:10.2196/29151)
Supplement: Multimedia Appendix 2 [file jmir_v23i8e29151_app2.docx]

**Multimedia Appendix 2.** Mobile Application Rating Scale scores for the 22 evaluated apps.

|  | MARS score dimension | | | | |
| --- | --- | --- | --- | --- | --- |
| Apps | Engagement mean score | Functionality mean score | Aesthetics mean score | Information mean score | App quality mean score |
| Bebe+ | 3.8 | 3.9 | 4.2 | 4.0 | 4.0 |
| iMamma | 3.5 | 3.6 | 3.7 | 3.5 | 3.5 |
| La mia gravidanza (Aleksei Neiman) | 2.5 | 4.3 | 3.3 | 2.1 | 3.0 |
| Autosvezzamento ricette veloci | 2.2 | 4.8 | 3.5 | 2.9 | 3.3 |
| Gravidanza+ | 3.5 | 4.5 | 4.0 | 3.9 | 3.9 |
| Mamma 2.0 | 1.6 | 2.5 | 1.8 | 1.8 | 1.9 |
| Vera mamma | 3.6 | 4.1 | 3.3 | 3.7 | 3.7 |
| Yoga in Gravidanza (la guida) | 3.4 | 3.9 | 4.2 | 3.3 | 3.7 |
| VITA: prodotti in gravidanza | 3.9 | 3.3 | 3.8 | 3.6 | 3.7 |
| Dalla nascita | 2.9 | 4.5 | 3.6 | 3.8 | 3.7 |
| Gravidanza Sprout | 3.5 | 3.6 | 3.8 | 2.9 | 3.5 |
| Mamma in salute | 4.0 | 4.9 | 4.3 | 4.8 | 4.5 |
| Calendario WomanLog Baby | 2.4 | 2.9 | 2.3 | 2.3 | 2.5 |
| La mia gravidanza (Doctissimo) | 4.1 | 4.4 | 4.0 | 3.5 | 4.0 |
| Non Da Sola | 2.5 | 2.8 | 2.8 | 3.7 | 2.9 |
| iBimbo | 2.4 | 4.4 | 3.7 | 2.4 | 3.2 |
| SOS bimbi | 2.3 | 4.5 | 4.0 | 3.8 | 3.6 |
| Happy mamma | 3.0 | 4.4 | 3.7 | 4.0 | 3.7 |
| Mustela per me | 3.1 | 3.6 | 3.9 | 2.8 | 3.3 |
| Seimammaeuganea | 2.9 | 4.1 | 3.7 | 3.6 | 3.6 |
| Pregnancy | 2.6 | 3.7 | 2.4 | 1.7 | 2.6 |
| eMyBaby | 2.0 | 3.7 | 2.9 | 2.2 | 2.7 |
